# Neoadjuvant Therapy in Resectable Advanced Melanoma: Swiss Real-World Data

**DOI:** 10.3390/cancers18010098

**Published:** 2025-12-28

**Authors:** Ann-Kathrin Blumenröther, Yongxing Fang, Tamara El Saadany, Egle Ramelyte, Omar Hasan Ali, Thomas Kündig, Daniela Mihic-Probst, Sarah Steiner, Alexander Maurer, Reinhard Dummer, Joanna Mangana, Lara V. Maul

**Affiliations:** 1 Department of Dermatology, University Hospital Zurich, University of Zurich, 8091 Zurich, Switzerland; ann-kathrin.blumenroether@usz.ch (A.-K.B.); omar.hasanali@usz.ch (O.H.A.); johanna.mangana@usz.ch (J.M.); 2 Faculty of Medicine, University of Zurich, 8032 Zurich, Switzerland; 3 Department of Pathology and Molecular Pathology, University Hospital Zurich, University of Zurich, 8091 Zurich, Switzerland; 4 Department of Nuclear Medicine, University Hospital Zurich, University of Zurich, 8091 Zurich, Switzerland

**Keywords:** melanoma, neoadjuvant, immune checkpoint inhibition, NADINA, SWOG S1801, pathologic response, radiologic response, mucosal

## Abstract

**Background/Objectives**: Neoadjuvant and perioperative treatment regimens for melanoma have demonstrated significantly longer event-free survival (EFS) compared with adjuvant therapy in the NADINA and SWOG S1801 trials. While these studies yielded promising results, real-world effectiveness and safety remain to be clarified. **Methods**: We performed a retrospective, real-world study of all patients with advanced, resectable cutaneous or mucosal melanoma stage III/IV who received neoadjuvant treatment at the Department of Dermatology, University Hospital Zurich, Switzerland between April 2023 and September 2025. Primary endpoints were pathologic and radiologic response, EFS, recurrence-free survival (RFS), and safety. **Results**: In total, 31 patients were analyzed (52% female; median age 65 years), including 5 patients without lymph node involvement. Eighteen patients (58%) with cutaneous melanoma received neoadjuvant immunotherapy according to the NADINA protocol, three patients (10%) with mucosal melanoma received ipilimumab (1 mg/kg) and nivolumab (3 mg/kg), and ten patients (32%) were treated according to the SWOG S1801 protocol. A major pathologic response (MPR) was achieved in 12 of 31 patients (38%) overall, including 5 of 18 (28%) in the NADINA cohort, 6 of 10 (60%) in the SWOG S1801 cohort, and 1 of 3 (33%) in the mucosal cohort. We observed a pathologic partial response (pPR) in 7 of 31 patients (23%) overall, including 6 of 18 (33%) in the NADINA cohort and 1 of 3 (33%) in the mucosal cohort. A pathologic non-response (pNR) was seen in 9 of 31 patients (29%) overall, including 5 of 18 (28%) in the NADINA cohort, 3 of 10 (30%) in the SWOG S1801 cohort, and 1 of 3 (33%) in the mucosal cohort. Among all patients without lymph node involvement, 1 of 5 achieved MPR (20%), 2 had pPR (40%), and 2 showed pNR (40%). At data cutoff (median follow-up, 9.2 months), the 9-month EFS was 77% in the NADINA cohort, 74% in the SWOG S1801 cohort, and 33% in the mucosal cohort. In the whole cohort, 6-month RFS for the subgroups of MPR, pPR and pNR was 72.9%, 85.7% and 72.9%. Radiologic response evaluation with FDG-PET/CT after neoadjuvant therapy correlated significantly with pathologic response (*p* = 0.02). No patient with complete metabolic response (CMR) or partial metabolic response (PMR) recurred until data cutoff. In total, 6 of 31 patients (19%) showed stable metabolic disease (SMD), and 8 of 31 patients (26%) showed progressive metabolic disease (PMD). The 6-month RFS in the subgroups of SMD and PMD was 62.5% in each case. Adverse events (AEs) of grade 3 or higher were reported in 13 of 31 patients (42%) in the total real-world cohort, 8 of 18 in the NADINA cohort (44%), 2 of 10 (20%) in the SWOG S1801 cohort (20%), and 3 of 3 (100%) in the mucosal cohort. The most frequent grade 3/4 toxicities were immune-related (ir) Colitis (*n* = 3, 10%), irHepatitis (*n* = 2, 6%) and irMyocarditis (*n* = 2, 6%). **Conclusions**: Neoadjuvant immunotherapy is effective in real-world practice with a similar safety profile as shown in the clinical studies. Nevertheless, MPR rates in the NADINA real-world cohort were lower compared to the phase III trial. Larger multicenter studies are needed to validate our findings and to better understand response patterns, even in patients without lymph node involvement and in rare melanoma subtypes.

## Simple Summary

Neoadjuvant immunotherapy has shown significant improvements in event-free survival (EFS) in recent phase II and III clinical trials. As trial populations are highly selective, they may not reflect the heterogeneity of patients encountered in clinical practice, especially those with comorbidities or rare melanoma subtypes. To address this gap, we conducted a retrospective real-world analysis of patients with advanced resectable melanoma treated with neoadjuvant immunotherapy at a tertiary referral center. We assessed pathological and radiological response, EFS and recurrence-free survival (RFS), and treatment-related toxicities. Our findings indicate meaningful clinical benefit but with lower response rates compared to pivotal trials, especially in patients with no lymph node involvement. Furthermore, this study raises the important question of how to optimize treatment strategies in high-risk subtypes such as mucosal melanoma.

## 1. Introduction

Patients with resectable stage III or IV melanoma (AJCC 8th edition 2017 [1]) remain at considerable risk for recurrence despite complete surgical resection of metastases and subsequent adjuvant therapy. The introduction of adjuvant therapy with immune checkpoint inhibitors (ICIs) or BRAF/MEK inhibitors has substantially enhanced outcomes in this setting. Pembrolizumab improved 7-year recurrence-free survival (RFS) to 50% compared with 36% for placebo [2], nivolumab achieved a 9-year RFS of 44% versus 37% for ipilimumab in stage IIIB-C/IV disease [3], and dabrafenib plus trametinib prolonged 10-year RFS to 48% compared with 32% for placebo [4]. The final results of the COMBI-AD trial demonstrated a significant overall survival (OS) benefit in patients with BRAF V600E–mutated melanoma, but not in the overall study population. To date, no OS data have been published on adjuvant ICIs.

The foundation of the neoadjuvant approach in the treatment of melanoma was established by Blank et al. in a small randomized controlled trial [5]. Ten patients were treated in the adjuvant setting with the anti-CTLA-4 antibody ipilimumab 3 mg/kg and anti-PD-1 antibody nivolumab 1 mg/kg for up to four courses following complete regional lymph node dissection (CLND), and ten patients received in a neoadjuvant setting two courses of ipilimumab/nivolumab using the same regimen prior to CLND and two courses thereafter. They observed a 2-year RFS of 80% in the neoadjuvant arm compared with 60% in the adjuvant arm. Pathological responses were achieved in 7/9 (78%) patients in the neoadjuvant arm. None of these patients had relapsed at data cutoff (median follow-up, 25.6 months). Importantly, neoadjuvant ipilimumab/nivolumab was associated with a greater expansion of tumor-resident T cell clones compared with adjuvant administration.

The OPACIN-neo trial [6] randomized 86 patients with stage IIIB/C disease [1] to different dosing regimens of ipilimumab and nivolumab. Across the treatment arms, pathologic response rate ranged from 65% to 80%, with grade 3/4 toxicities occurring in 20% to 50% of patients. Patients who achieved a pathologic response had an impressive 2-year RFS of 97% compared with only 36% in non-responders. Building on these results, the PRADO study [7] investigated a response-adapted surgical approach in 99 patients with stage IIIB/C melanoma. Following two cycles of “flip-dose” ipilimumab/nivolumab, patients with complete or near-complete response (65%) in their index lymph node omitted CLND, and adjuvant therapy maintained a 2-year RFS of 93%. In contrast, those with partial or absent pathologic responses underwent more extensive local and systemic treatment, with lower RFS rates of 64% and 71%, respectively.

Recently, the phase III NADINA trial [8] provided solid evidence supporting the benefit of neoadjuvant therapy. Among 423 patients with macroscopic stage III melanoma, two cycles of neoadjuvant ipilimumab/nivolumab significantly improved 2-year event-free survival (EFS) to 77% compared with 56% for standard surgery followed by adjuvant nivolumab [9]. EFS was defined as the time from randomization to progression to unresectable melanoma before surgery, disease recurrence, or death due to melanoma or treatment. In the neoadjuvant arm, adjuvant systemic therapy and radiotherapy were reserved for patients who did not achieve a major pathological response (MPR).

Likewise, the randomized phase II SWOG S1801 trial demonstrated that three cycles of neoadjuvant pembrolizumab followed by surgery and subsequent adjuvant pembrolizumab yielded a 3-year EFS of 68%, compared with 55% in patients who received pembrolizumab exclusively in the adjuvant setting [10,11]. It is important to note that EFS in this trial was defined slightly differently as the time from randomization to disease progression, treatment-related toxicity precluding surgery, the inability to achieve complete resection, surgical complications or treatment-related toxicity preventing initiation of adjuvant therapy within 84 days after surgery, recurrence of melanoma after surgery, or death from any cause. Postoperative radiotherapy was also eligible.

A perioperative regimen combining nivolumab with the anti-LAG-3 antibody relatlimab achieved 57% pathologic complete responses (pCR) and a 2-year EFS of 81% [12]. In the phase III PIVOTAL trial, neoadjuvant intralesional daromun (a combination of two antibody cytokine fusions, L19IL2 and L19TNF) showed significantly longer RFS than surgery alone, with a median RFS of 16.7 months vs. 6.8 months [13].

While clinical trials highlight the efficacy of neoadjuvant therapy, their stringent eligibility criteria often exclude patients with comorbidities, reduced performance status, atypical disease presentations, or rare melanoma subtypes. As a result, trial findings may overestimate efficacy and underestimate treatment-related toxicities. Toxicities are particularly concerning given the curative intent of neoadjuvant therapy, as prolonged delays to surgery or permanent organ damage could negate survival benefits.

Consequently, real-world data are needed to characterize the efficacy and safety profile of neoadjuvant immunotherapy across diverse patient populations to guide risk-adapted strategies. To address this need, we conducted a retrospective, monocentric real-world analysis of patients with advanced resectable melanoma treated with neoadjuvant immunotherapy according to the NADINA and SWOG S1801 protocols.

## 2. Materials and Methods

### 2.1. Study Design and Population

This single-center retrospective study included all patients with resectable advanced melanoma who received neoadjuvant treatment at the Department of Dermatology, University Hospital Zurich, between April 2023 and September 2025 (data cutoff 1 September 2025). Patients were treated either with ipilimumab/nivolumab (Bristol Myers Squibb, Princeton, NJ, USA) according to the NADINA protocol [8] or pembrolizumab (Merck Sharp & Dohme, Kenilworth, NJ, USA) according to the SWOG S1801 protocol [10]. Eligible patients had stage III cutaneous melanoma including melanoma of unknown primary or stage III/IV mucosal melanoma. We included patients with lymph node metastasis, in-transit metastasis, locally advanced primary tumors, or a combination of the above. Lymph node involvement was not mandatory. Patients with prior adjuvant therapy were eligible.

Patients in the NADINA group received two neoadjuvant cycles of ipilimumab (80 mg) and nivolumab (240 mg) every three weeks. Patients with mucosal melanoma were given weight-adjusted treatment with two neoadjuvant cycles of ipilimumab (1 mg/kg) and nivolumab (3 mg/kg). Patients in the SWOG S1801 cohort received three neoadjuvant cycles of pembrolizumab (200 mg) every three weeks. One to two weeks after the final neoadjuvant cycle, patients underwent FDG-PET/CT (2-[^18^F]-fluoro-deoxy-D-glucose-positron emission tomography/computed tomography) scans to assess radiologic response, followed by surgery except in cases with non-resectable progression. Surgical procedures primarily included lymphadenectomies, often combined with resections of in-transit metastases or re-excisions of the primary tumor with safety margins; less commonly, isolated excisions of in-transit metastases or primary tumors were performed.

According to the NADINA protocol, patients with a complete or near-complete pathological response (summarized as MPR; ≤10% residual tumor) did not receive adjuvant therapy. Patients with pathologic partial (pPR) or non-response (pNR) continued the adjuvant therapy for up to one year. For BRAF-V600E-mutated patients, a switch to dabrafenib/trametinib was eligible. Adjuvant radiotherapy was allowed, except for patients who had an MPR. Initially, all SWOG S1801 patients received adjuvant pembrolizumab irrespective of response, according to the protocol. Based on the results of the NADINA trial, the administration of adjuvant therapy was likewise adapted according to pathological response from the beginning of 2025. Treatment was discontinued in case of progression, unacceptable toxicity, death, or withdrawal of consent. Immune-related adverse events (irAEs) were treated in accordance with internationally established guidelines [14].

### 2.2. Endpoints and Assessment

The primary endpoint was EFS, defined as in the NADINA trial [8] as the time from initial staging to melanoma progression to irresectable disease, melanoma recurrence, or death due to melanoma or treatment. Secondary endpoints included RFS, defined as recurrence after surgery or death, OS, distant metastasis–free survival (DMFS), pathological and radiological response, their correlation, and safety. Data for patients who did not have an event were censored at the date of the last available imaging. Pathological response was assessed according to International Neoadjuvant Melanoma Consortium (INMC) criteria by the University Hospital Zurich Pathology Department and classified as pCR (0% viable tumor), near-pCR (> 0 ≤ 10%; pathologic near-complete response), pPR (> 10 ≤ 50%), or pNR (>50%) [15]. Radiologic response was evaluated per imPERCIST5 (immunotherapy-modified Positron Emission Response Criteria in Solid Tumors up to Five Lesions) [16,17,18,19] by two independent nuclear medicine physicians of the Department of Nuclear Medicine of the University Hospital Zurich. In the event of a discrepancy, a consensus was achieved through consensus discussion. Radiologic response was categorized as complete metabolic response (CMR), partial metabolic response (PMR), stable metabolic disease (SMD), or progressive metabolic disease (PMD) based on the change in the SUVpeak (∆SUV*_peak_*, peak standardized uptake value) in % from the baseline. Adverse events were graded using the NCI Common Terminology Criteria for Adverse Events (CTCAE), version 5.0 [20].

### 2.3. Statistical Analysis

Patient demographics and clinical characteristics were described as median or mean (range) for continuous variables and frequency (proportion) for categorical data. EFS and RFS were calculated using the Kaplan–Meier method. The RFS was stratified by response category within each modality (pathologic and radiologic response). Group differences in EFS and RFS were tested using the log-rank test. No confidence intervals were calculated for groups without events. Associations between ∆SUV*_peak_* and pathologic response were assessed using the Kruskal–Wallis test. All statistical analyses were performed with GraphPad Prism (version 10.5.0). A *p*-value below 0.05 was considered statistically significant.

## 3. Results

### 3.1. Patient and Treatment Characteristics

We included 31 patients with advanced resectable melanoma (52% female, median age 65 years) (Table 1). Eighteen patients with cutaneous melanoma (58%) received dual immunotherapy with ipilimumab/nivolumab (2 infusions of 80/240 mg, once every 3 weeks). Three patients (10%) with mucosal melanoma received a weight-adjusted dose of 1 mg/kg ipilimumab and 3 mg/kg nivolumab (2 infusions, once every 3 weeks). Ten patients with cutaneous melanoma (32%) received anti-PD-1 monotherapy with pembrolizumab (3 infusions of 200 mg, once every 3 weeks). Additional information about patient and treatment characteristics can be found in Table A1.

### 3.2. Pathological and Radiological Response Rates and Associations with Each Other

**Figure 1:**
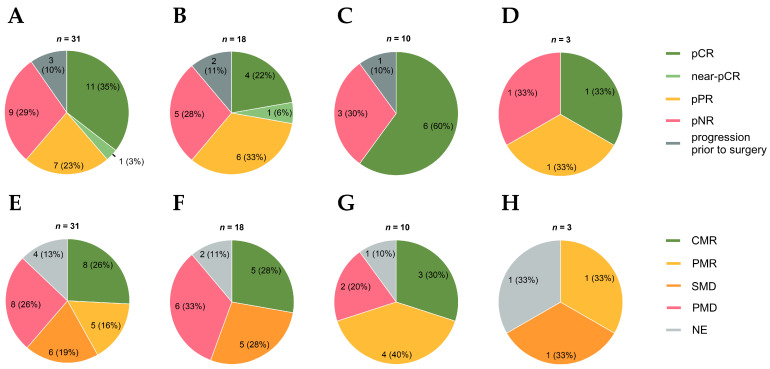
Pathologic (**A**–**D**) and radiologic (**E**–**H**) response rates. Pathologic response rates for (**A**) the total cohort, (**B**) the NADINA cohort, (**C**) the SWOGS 1801 cohort, and (**D**) the mucosal cohort. pCR, pathologic complete response; Near-pCR, near-complete pathologic response; pPR, pathologic partial response; pNR, pathologic non-response. Radiologic response rates for (**E**) the total cohort, (**F**) the NADINA cohort, (**G**) the SWOG S1801 cohort, and (**H**) the mucosal cohort. CMR, complete metabolic response; PMR, partial metabolic response; SMD, stable metabolic disease; PMD, progressive metabolic disease; NE, not evaluable (melanoma manifestation not detectable by FDG-PET/CT).

Of 31 patients, 28 (90%) underwent surgery and were assessable for RFS, while 3 (10%) did not undergo surgery due to progression to unresectable disease (Table A1). In the total real-world cohort, we observed an MPR in 12 patients (39%), a pPR in 7 (23%), and a pNR in 9 (29%). In the NADINA cohort, we detected an MPR in 5 of 18 patients (28%), a pPR in 6 (33%), and a pNR in 5 (28%). In the SWOG S1801 cohort, an MPR was achieved in 6 of 10 patients (60%) and a pNR in 3 (30%). In the mucosal cohort, we observed an MPR in 1 of 3 patients (33%), a pPR in 1 (33%), and a pNR in 1 (33%). Among patients without lymph node involvement, 1 of 5 achieved an MPR (20%), 2 a pPR (40%), and 2 a pNR (40%) (Figure 1).

Thirty-one patients were assessed for radiologic response with FDG-PET using imPERCIST5 criteria [17]. Some 4 of 31 patients (13%) were not evaluable as the lesions were not detectable with the initial staging FDG-PET/CT. In the total cohort, eight patients had a CMR (26%), five a PMR (16%), six SMD (19%) and eight PMD (26%) (Figure 1). Among patients classified with CMR, the majority also exhibited MPR (71%, 5/7) (Table A2). Among patients with PMR, three patients (60%, 3/5) had an MPR.

**Figure 2:**
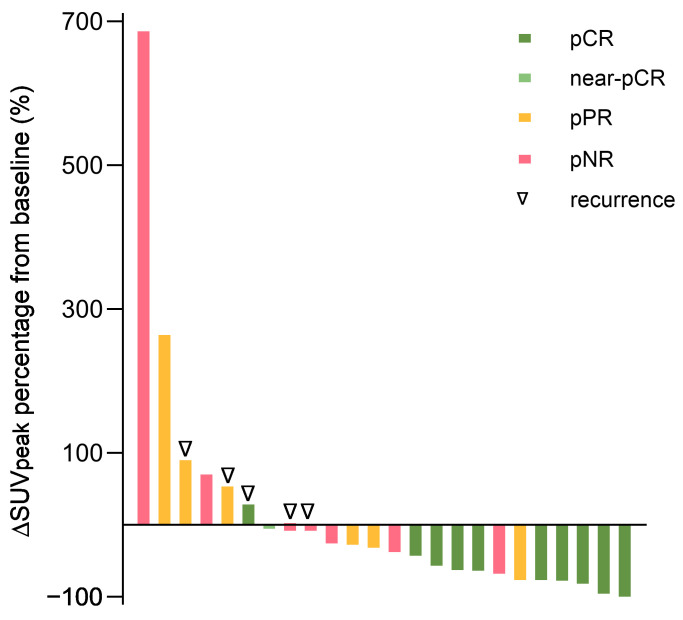
Waterfall plot by ∆SUV_peak_ associated with pathologic response, *n* = 24 (the reason for the reduced sample size is that patients who progressed prior to surgery and therefore had no pathologic assessment and patients without measurable tumors in radiologic imaging are not included). pCR, pathologic complete response; near-pCR, near-complete pathologic response; pPR, pathologic partial response; pNR, pathologic non-response; SUV, standardized uptake value.

Most of the patients with progressive metabolic disease (PMD) were pathologically partial or non-responsive, although one patient (17%, 1/6) achieved MPR. There was a significant correlation between ∆SUV*_peak_* and pathologic response (*p* = 0.02) (Figure 2). In the NADINA cohort, 5 of 18 patients (28%) showed CMR, 5 SMD (28%), and 6 PMD (33%) (Figure 1). In the SWOG S1801 cohort, we detected CMR in 3 of 10 patients (30%), PMR in 4 (40%), and PMD in 2 (20%). In the mucosal cohort, we observed PMR in one of three patients (33%), SMD in one (33%), and one patient was not evaluable because the lesion was not detectable on the initial staging FDG-PET/CT.

### 3.3. Event-Free Survival

**Figure 3:**
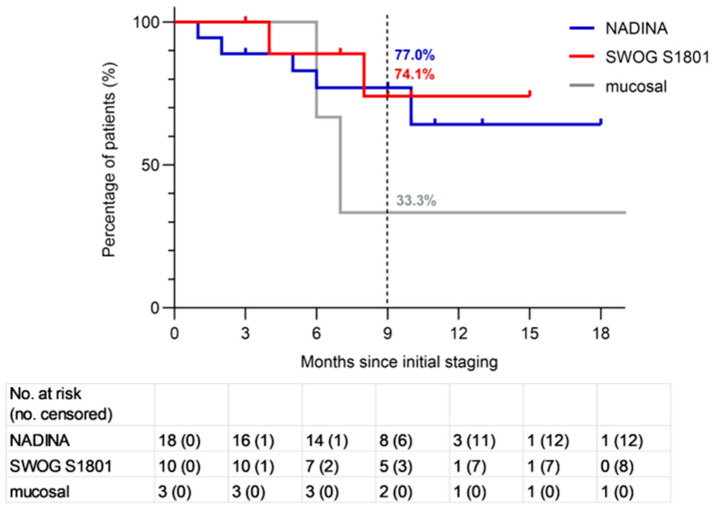
Kaplan–Meier curves for event-free survival (EFS) for the different real-world cohorts (NADINA, SWOG S1801, mucosal). The landmark 9-month EFS rate was 77% (95% CI 57.2% to 96.8%) in the NADINA real-world cohort; 74.1% (95% CI 42.6% to 100%) in the SWOG S1801 real-world cohort; and 33.3% (95% CI 0% to 86.6%) in the mucosal real-world cohort.

At the time of data cutoff (1 September 2025), the median duration of follow-up was 9.2 months (interquartile range 6.2 to 10.9); in the NADINA cohort, it was 8.9 months (interquartile range 5.8 to 10.8), and it was 9.1 months (interquartile range 7.1 to 9.9) in the SWOG S1801 cohort. It was 14 months (interquartile range 10.6 to 21.6) in the mucosal cohort. A total of nine events (progression, recurrence, or death from melanoma or treatment) occurred in the total population, including five in the NADINA, two in the SWOG S1801, and two in the mucosal cohort. The landmark 9-month EFS rate was 77% (95% CI 57.2% to 96.8%) in the NADINA cohort and 74.1% (95% CI 42.6% to 100%) in the SWOG S1801 cohort (Figure 3). The mucosal cohort showed a 9-month EFS rate of 33.3% (95% CI 0% to 86.6%). Of the 12 patients with BRAF-V600E mutations, 3 had a pNR and received adjuvant targeted therapy. None of these patients had recurred at data cutoff. At the time of analysis, data for OS and DMFS remain immature.

### 3.4. Recurrence-Free Survival and Correlation with Pathological and Radiological Response

**Figure 4:**
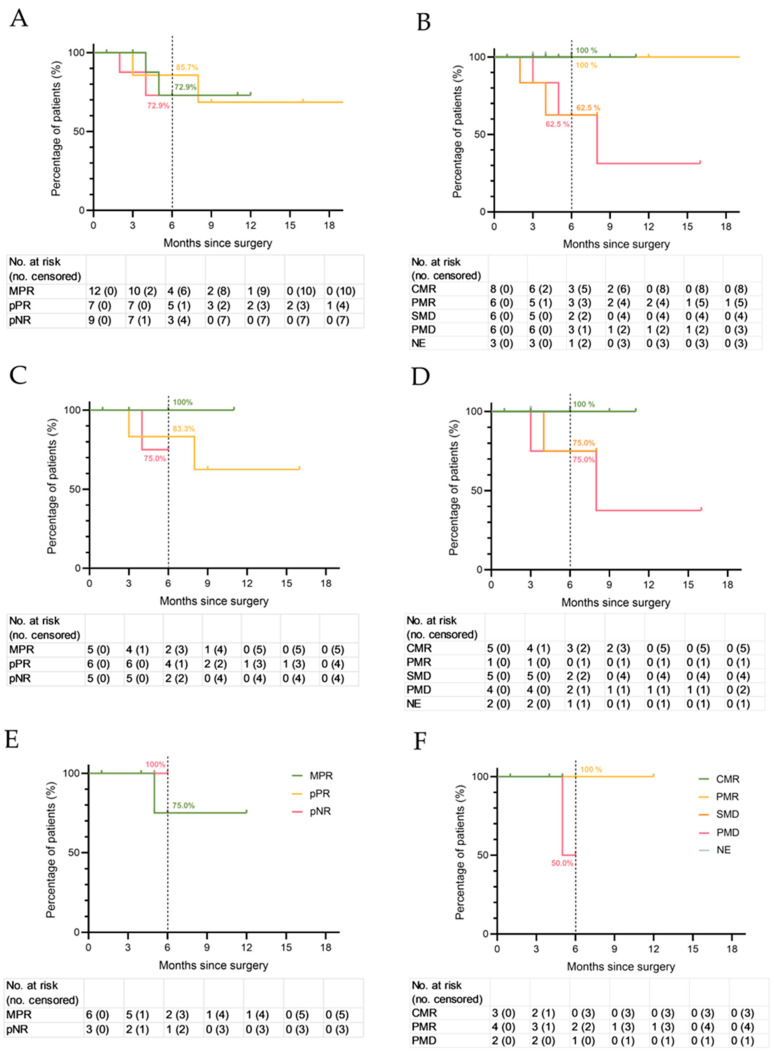
Recurrence-free survival (RFS) by pathologic (**A**,**C**,**E**) and radiologic (**B**,**D**,**F**) response for (**A**,**B**) the total real-world cohort, (**C**,**D**) the NADINA real-world cohort, and (**E**,**F**) the SWOG S1801 real-world cohort. MPR, major pathologic response; pPR, pathologic partial response; pNR, pathologic non-response. CMR, complete metabolic response; PMR, partial metabolic response; SMD, stable metabolic disease; PMD, progressive metabolic disease; NE, not evaluable (melanoma manifestation not measurable by radiologic imaging).

In the total cohort, the 6-month RFS rates for patients with MPR, pPR, and pNR were 72.9% (95% CI 40.6% to 100%), 85.7% (95% CI 59.8% to 100%), and 72.9% (95% CI 62.6% to 100%), respectively (Figure 4A). In the NADINA cohort, the 6-month RFS rates for patients with MPR, pPR, and pNR were 100%, 83.3% (95% CI 20.7% to 100%), and 75% (95% CI 33.6% to 100%), respectively (Figure 4C). In the SWOG S1801 cohort, we observed 6-month RFS rates of 75% (95% CI 33.6% to 100%) for patients with MPR and 100% for patients with pNR (*n* = 1) (Figure 4E).

In the whole cohort, the 6-month RFS rates for patients with CMR, PMR, SMD and PMD were 100%, 100%, 62.5% (95% CI 20.7% to 100%), and 62.5% (95% CI 20.7% to 100%), respectively (Figure 4B). The four radiologically non-evaluable patients had a 6-month RFS of 100%. In the NADINA cohort, the 6-month RFS rates for patients with CMR, PMR, SMD and PMD were 100%, 100%, 75.0% (95% CI 32.6% to 100%), and 75.0% (95% CI 32.6% to 100%), respectively (Figure 4D). In the SWOG S1801 cohort, the 6-month RFS rates for patients with CMR, PMR and PMD were 100%, 100%, and 50% (95% CI 0% to 100%), respectively (Figure 4F).

In the mucosal cohort, one patient, who showed PMR and pPR, remains in complete remission after 29 months. This patient received another two doses of ipilimumab/nivolumab in the adjuvant setting before switching to nivolumab monotherapy. One patient with pNR and SMD had a local recurrence after two months. One patient with pCR was in remission until the patient died from an irAE as described below.

### 3.5. Safety

Adverse events (AEs) of any cause of grade 3 or higher were reported in 13 of 31 patients (42%) in the total real-world cohort, 8 of 18 (44%) in the NADINA cohort, 2 of 10 (20%) in the SWOG S1801 cohort, and 3 of 3 (100%) in the mucosal cohort (Table 2). AEs of grade 3 or higher that were related to systemic treatment occurred in 10 of 31 patients (32%) in the total cohort, 5 of 18 patients (28%) in the NADINA cohort, 2 of 10 patients (20%) in the SWOG S1801 cohort, and 3 of 3 (100%) in the mucosal cohort. Surgery-related AEs of grade 3 or higher occurred in 3 of 31 patients (10%) in the total cohort and 3 of 18 (17%) in the NADINA cohort. We reported no surgery-related AEs of grade 3 or higher in the SWOG S1801 or mucosal cohort.

In the whole cohort, 7 of 31 patients (23%) had an AE of grade 3 or higher that was related to systemic treatment within the first 12 weeks and was attributable solely to the neoadjuvant treatment. Some 4 of 18 patients (22%) in the NADINA cohort, 1 of 10 (10%) in the SWOG S1801 cohort, and 2 of 3 (67%) in the mucosal cohort had a grade 3 or higher AE attributable to the neoadjuvant cycles of immunotherapy. In 10% of patients, surgery was postponed due to AEs, with a median delay of two weeks.

We observed a varying probability of occurrence across the spectrum of AEs of grade 3 or higher with a higher rate for rare immune-related entities like myocarditis or encephalitis (Table 2). In the mucosal cohort, one treatment-related death occurred. The patient developed immune-related myocarditis after a single neoadjuvant infusion of ipilimumab/nivolumab and was treated with high-dose steroids and intravenous immunoglobulins. The patient died 6 months later from a recurrent myocarditis and non-ST-elevation myocardial infarction (NSTEMI) after refusing invasive ischemia diagnostics due to declining condition.

## 4. Discussion

Here, we present the first neoadjuvant real-world cohort of advanced resectable melanoma treated in line with both the NADINA and the SWOG S1801 protocols according to individual patient characteristics.

The 9-month EFS rates observed in our cohorts (77% and 74%, respectively) mirror those of the pivotal trials, underscoring the feasibility and robustness of neoadjuvant approaches in routine clinical practice.

Nonetheless, relevant differences emerged: lower MPR rates—especially in the NADINA subgroup and among node-negative patients—and a distinct pattern of adverse events highlight the complexity of translating trial outcomes into real-world settings.

### 4.1. Pathologic Response

We observed a 39% MPR rate in the total real-world cohort, 60% in the SWOG S1801 cohort, 28% in the NADINA cohort, and 33% in the mucosal cohort. Our results align closely with the 51% reported in the SWOG S1801 trial [21]. In contrast, the results in the NADINA cohort differ substantially from the pivotal trial which showed an MPR rate of 59% [8]. Other real-world studies reported MPR/pCR rates of 43–50% [22,23]. Importantly, in our NADINA cohort, inferior radiological responses were also noted compared to the total study population. Patient characteristics, including ECOG performance status, age, and prior adjuvant therapy may have contributed to the poorer outcomes. Furthermore, our NADINA cohort and mucosal cohort exhibited a lower TMB than our SWOG S1801 cohort (19 mut/Mb vs. 12 mut/Mb vs. 29 mut/Mb). As TMB represents an independent predictor of response to immunotherapy, this difference is noteworthy [24,25]. Differences in pathological analysis also could have contributed to diverting MPR rates. In the NADINA trial, pathological response was evaluated only in the lymph node specimens, even if in-transit metastases were excised [8]. In the SWOG S1801 trial and in our study, pathological response was evaluated across all specimens of a patient, which may have led to important differences in the individual assessment of patients’ responses. Another factor may be the presence of lymph node involvement, which constituted a mandatory inclusion criterion in the NADINA trial, but not in the SWOG S1801 trial. Among the 5 patients in our cohort without lymph node involvement, we observed an MPR in 1 patient (20%, 1/5), two pPR (40%, 2/5), and two pNR (40%, 2/5). In the NEO-MEL study, MPR rates also differed by this factor, with 16% (3/19) for isolated in-transit metastases and 49% (39/80) for isolated lymph node disease [26]. An important topic to discuss in this context is tumor heterogeneity [27]. The immune phenotype distribution is significantly different in metastatic samples compared to primary melanomas [28,29]. Primary tumors often show more lymphoid aggregates and histologic regression than metastases, suggesting a stronger initial immune presence, but response in lymph nodes may be more relevant for early relapse.

Out of these discrepancies rise three questions. Firstly, is the immunogenicity of primary tumors and subcutaneous metastases sufficient to elicit a meaningful preoperative response to immunotherapy, justifying a neoadjuvant approach over direct excision followed by adjuvant therapy? Secondly, do the pathological changes observed in primary tumors or subcutaneous metastases reflect the patient’s anti-tumor response and thus help to guide treatment decisions, such as the need for adjuvant therapy? Thirdly, is the pathologic response in a lymph node a better surrogate marker for response and relapse compared to subcutaneous metastases or primary tumors? Further translational studies are needed to analyze pathologic alterations and immunologic response patterns in the different tissues to clarify the efficiency of neoadjuvant therapy for patients with solely in-transit metastasis and locally advanced primary tumors.

### 4.2. Correlation of Pathologic and Radiologic Response

We observed a strong concordance between pathological response and radiological response measured by FDG-PET per imPERCIST5, and ∆SUV*_peak_* was significantly associated with pathologic response. Furthermore, radiologic response was a strong predictor of RFS, as no patient with CMR and PMR recurred until data cutoff. Our findings support that radiologic imaging can serve as a reliable surrogate marker of treatment efficacy in the neoadjuvant setting. Our data align with the results of Zhou et al., who demonstrated a significant correlation of pathological and radiological responses measured by PET-CT as well as survival outcome in 115 patients [16]. The preoperative radiologic response could serve as a complementary biomarker to personalize surgical decision making. For instance, a CMR might allow for a less extensive surgical approach. As demonstrated in the PRADO trial [7], selective resection of the index lymph node instead of complete lymphadenectomy can reduce postoperative morbidity and lower healthcare costs. Conversely, in cases showing PMD, escalation of therapy through broader surgical interventions, adjuvant radiotherapy, or intensified systemic therapy may be warranted.

### 4.3. Event-Free Survival

The estimated 9-month EFS was 77% in the NADINA cohort and 74.1% in the SWOG S1801 cohort. These results are consistent with the SWOG S1801 trial, which reported an estimated 12-month EFS of 72% and no additional events up to 24 months [10]. By contrast, the NADINA trial reported a higher 12-month EFS of 83% [8], and the update presented at ESMO 2025 showed a 24-month EFS of 77% in the neoadjuvant arm [9]. This apparent advantage in the NADINA trial compared to our real-world data may be explained in part by its stricter eligibility criteria, such as inclusion limited to patients with ECOG 0–1 and mandatory lymph node involvement, as discussed above. Because the observation time in our study is relatively short (median 9.2 months; interquartile range 6.2 to 10.9), longer follow-up is required to evaluate the long-term outcome of the different neoadjuvant treatment regimens. It should be emphasized that a direct comparison between the SWOG S1801 and the NADINA real-world cohort lacks scientific validity due to highly differing patient populations. Therefore, no conclusions regarding a favorable regimen can be drawn from our results. The choice between the NADINA and the SWOG S1801 protocols, if both are reimbursable, should be individualized, taking all patient-specific factors into account and decided by consensus within an interdisciplinary tumor board. Factors influencing the decision in favor of a combined ICI in our cohort included younger age, few comorbidities, high tumor load, low TMB, elevation of LDH and s100, pretreatments, and high-risk subtypes such as mucosal or acral lentiginous melanoma.

### 4.4. Recurrence-Free Survival

In our total real-world cohort, we observed a 6-month RFS of 72.9% for patients with MPR, 85.7% for pPR, and 72.9% for pNR. As differences between groups were less pronounced than expected, the underlying reason remains unclear. Possible explanations may lie in distinct pathologic and immunologic response patterns of in-transit metastasis or primary tumors, especially in mucosal locations (as discussed elsewhere in this manuscript); limited subgroup size; the highly heterogeneous patient cohort; and the relatively short follow-up period, especially in the subgroup of patients who achieved MPR.

The RFS categorized by pathologic response in the NADINA cohort mirrors the results of the pivotal trial with a 6-month RFS of 100% for patients with MPR, 83% for pPR, and 75% for pNR. Our results support the hypothesis that it is safe to omit adjuvant therapy in cases of MPR after neoadjuvant dual ICI, as none of the patients with MPR in the NADINA cohort had a recurrence.

In our SWOG S1801 cohort, we observed a 6-month RFS of 75% for patients with MPR and 100% for pNR. In our study, pathological response was also used to guide the decision on administration of adjuvant treatment for most patients in the SWOG S1801 cohort, even though the original clinical trial did not report on this strategy. Four patients with MPR in our SWOG S1801 cohort did not receive adjuvant therapy, and only one recurrence was observed. Notably, this BRAF-wildtype patient was ineligible for adjuvant immunotherapy due to immune-related encephalitis. In the Swedish real-world cohort of the NEO-MEL study, among the patients who had an MPR, 9/49 received no adjuvant treatment; their RFS was similar to patients who received adjuvant treatment [26]. While omission of adjuvant therapy after MPR following neoadjuvant anti-PD-1 monotherapy can be evaluated, further studies are warranted given the small sample sizes and limited follow-up.

### 4.5. Neoadjuvant Treatment in Mucosal Melanoma

In our mucosal cohort, we reported a low 9-month EFS rate of 33%. These findings should be interpreted with caution because of the small sample size (*n* = 3). Ho et al. reported that neoadjuvant immunotherapy represents an effective and feasible strategy for mucosal melanoma. They described a 3-year EFS of 29% and 3-year OS rate of 55% for patients treated with anti-PD-1 ± anti-CTLA-4 antibodies in a neoadjuvant setting [30]. Given the rarity of this melanoma subtype, no established standard approach exists for resectable disease. Notably, in our cohort, one patient received two additional doses of ipilimumab/nivolumab in the adjuvant setting before switching to nivolumab monotherapy. This patient, who initially showed PMR and pPR, remains in complete remission after 29 months. Such cases highlight the potential value of intensified combination strategies—such as four doses of ipilimumab/nivolumab or use of the ipilimumab 3 mg/kg and nivolumab 1 mg/kg regimen—as possible approaches to counteract the lower response rates of mucosal melanoma to immunotherapy.

### 4.6. Safety

In our real-world cohort, grade ≥ 3 immune-related adverse events (irAEs) occurred in 32% of patients overall and in 28% of the NADINA real-world cohort, closely matching the 30% reported in the NADINA trial. In contrast, the SWOG S1801 real-world cohort showed a higher incidence than in the pivotal trial (20% vs. 7%), likely reflecting a less selected, older, and frailer patient population. Differences in AE reporting, supportive care, and treatment discontinuation patterns may also have contributed. Importantly, we observed rare, unexpected toxicities such as encephalitis and myocarditis, as well as one treatment-related death after a single infusion of ipilimumab/nivolumab, underscoring that severe immune-mediated toxicity can occur even after short treatment exposure. These findings emphasize the need for careful patient selection, early recognition, and proactive management of irAEs in real-world practice.

### 4.7. Access to Neoadjuvant Therapy

To date, neoadjuvant immunotherapy regimens are not yet approved by the European Medicines Agency or the U.S. Food and Drug Administration, and they remain unapproved in most countries, except for the Netherlands, Italy and Australia. Some national and international melanoma treatment guidelines already incorporated clear recommendations for the neoadjuvant treatment approach, such as the ESMO and SWISS guidelines [31,32]. Nevertheless, many patients lack access to these therapeutic advances. Hauschild et al. conducted an international survey among 104 melanoma specialists, revealing broad acceptance of both the SWOG S1801 and NADINA neoadjuvant regimens, with a slight preference for the perioperative scheme with pembrolizumab [33]. This preference likely reflects the easier reimbursement of the SWOG S1801 approach, since pembrolizumab is already authorized for one year of adjuvant therapy at the same dosage, with surgery merely deferred until after the third ICI administration. Notably, 89% of specialists supported the immediate introduction of neoadjuvant ICI therapy into standard clinical practice. Regarding safety, all surveyed experts (100%) deemed both NADINA and SWOG S1801 sufficiently safe for use in the majority of patients. Pivotal trials, our results, and further experiences in real-world settings [22,23,26] support the broader adoption of these regimens for advanced resectable melanoma.

### 4.8. Strengths and Limitations

This is the first real-world analysis showing patients treated in line with both the NADINA and the SWOG S1801 protocol according to individual patient characteristics. While clinical trials have established the foundation of the neoadjuvant treatment concept, our real-world study provides valuable insights into everyday clinical practice, encompassing an unselected and pretreated patient population as well as rare melanoma subtypes. Because of the limited sample size in the mucosal cohort, these results should be interpreted as a distinct case series. Furthermore, our total cohort included patients without lymph node involvement, a group excluded from the NADINA trial, thereby broadening the clinical spectrum of neoadjuvant therapy. Consequently, pathological assessment differed from that in NADINA and should be considered when interpreting response rates. In addition, the inhomogeneity of the patient cohort may have confounded the interpretation of treatment effects by introducing variability. Along with the single-center design, limited sample size, and relatively short follow-up, these factors limit the generalizability of our findings.

## 5. Conclusions

In summary, this real-world study supports the efficacy and safety of neoadjuvant ICI therapy in patients with resectable advanced melanoma, consistent with pivotal clinical trials. However, we observed lower MPR rates in the NADINA real-world cohort and a distinct spectrum of adverse events across our population. These discrepancies likely reflect broader inclusion criteria, including patients without lymph node involvement, those with prior adjuvant therapy, and an older and more comorbid patient population. Our findings underscore the need for biomarker-driven patient selection, as outcomes in unselected or vulnerable patients may diverge from trial populations. Given the limited sample size and heterogeneity, further multicenter studies are warranted to validate these results and better define optimal strategies, particularly for rare melanoma subtypes.

## Figures and Tables

**Table 1:** Patient and treatment characteristics *.

Characteristics	Total Real-World Cohort *n* = 31	NADINA Real-World Cohort *n* = 18	SWOG 1801 Real-World Cohort *n* = 10	Mucosal Real-World Cohort *n* = 3
Sex—no. (%)				
Female	16 (52)	7 (39)	6 (60)	3 (100)
male	15 (48)	11 (61)	4 (40)	0
Median age—years (range)	65 (31–95)	57 (31–73)	75 (64–95)	79 (49–87)
Median weight—kilogram (range)	79.5 (41–110)	83 (41–110)	74 (50–103)	65 (56–76)
Melanoma type—no. (%)				
Nodular	6 (19)	5 (28)	1 (10)	0
Superficial spreading	8 (26)	2 (11)	6 (60)	0
Acral lentiginous	3 (10)	3 (17)	0	0
Other cutaneous types £	7 (23)	5 (28)	2 (20)	0
Melanoma of unknown primary	4 (13)	3 (17)	1 (10)	0
Mucosal	3 (10)	0	0	3 (100)
Disease stage of cutaneous melanoma—no. (%) †				
IIIB	6 (19)	5 (28)	1 (10)	n.a.
IIIC	22 (71)	13 (72)	9 (90)	n.a.
Disease stage of mucosal melanoma—no. (%) ‡				
IIIC	2 (6)	n.a.	n.a.	2 (67)
IVA	1 (3)	n.a.	n.a.	1 (33)
TMB (mut/mb)—median (range)	19 (5–51)	19 (5–26)	29 (12–51)	12 (8–35)
BRAF-mutation status—no. (%)				
Wildtype	13 (42)	5 (28)	5 (50)	3 (10)
V600E	12 (39)	10 (56)	2 (20)	0
V600K	2 (6)	1 (6)	1 (10)	0
Other BRAF mut	3 (10)	2 (11)	1 (10)	0
Not measured	1 (3)	0	1 (10)	0
Previous sentinel lymph node biopsy—no (%)	8 (26)	4 (22)	4 (40)	0
Previous sentinel lymph node biopsy—no (%)	8 (26)	4 (22)	4 (40)	0
Indication for neoadjuvant treatment cutaneous melanoma—no. (%)				
Head and neck lymph node metastases	4 (13)	3 (17)	1 (10)	0
Axillary lymph node metastases	11 (35)	7 (39)	4 (40)	0
Inguinal lymph node metastases	7 (23)	6 (33)	0	1 (33)
ITMs only	3 (10)	1 (6)	2 (20)	0
ITMs with synchronous lymph node metastases	3 (10)	0	3 (30)	0
Primary	2 (6)	0	0	2 (67)
Primary with synchronous ITMs	1 (3)	1 (6)	0	0
Subcutaneous metastases (stage IV)	0	0	0	0
Other stage IV	0	0	0	0
Adjuvant systemic treatment—no. (%)	16 (52)	10 (56)	5 (50)	1 (33)
Nivolumab	7 (23)	7 (39)	0	0
2 doses of Ipilimumab/Nivolumab and followed by Nivolumab ¢	1 (3)	0	0	1 (33)
Pembrolizumab	5 (16)	0	5 (50)	0
Targeted therapy (Dabrafenib/Trametinib)	3 (10)	3 (17)	0	0
Adjuvant radiotherapy	5 (16)	2 (11)	2 (20)	1 (33)

Abbreviations: ITM: In-transit metastasis; * Percentages may not total 100 because of rounding; £ Other cutaneous types including dermal, desmoplastic, naevoid, spitzoid, non-classifiable and lentigo maligna melanoma; † Stages are defined according to the American Joint Committee on Cancer Staging Manual, 8th edition; ‡ Stages are defined according to the S3-Guideline Diagnosis, therapy and follow-up of melanoma Version 3.3; ¢ This patient with mucosal melanoma received two doses of ipilimumab/nivolumab before and two doses after surgery, as the NADINA protocol had not been published at that time point. The patient is in total remission until data cutoff.

**Table 2:** Safety *.

Safety	Total Real-World Cohort *n* = 31	NADINA Real-World Cohort *n* = 18	SWOG S1801 Real-World Cohort *n* = 10	Mucosal Real-World Cohort *n* = 3
Any AE	25 (81)	17 (94)	5 (50)	3 (100)
Any AE ≥ grade 3 †	13 (42)	8 (44)	2 (20)	3 (100)
Any irAE	23 (74)	15 (83)	5 (50)	3 (100)
Any irAE ≥ grade 3 †	10 (32)	5 (28)	2 (20)	3 (100)
Most frequent irAE ≥ grade 3 †				
Colitis	3 (10)	2 (11)	0	1 (33)
Myocarditis	2 (6)	1 (6)	0	1 (33)
Hepatitis	2 (6)	2 (11)	0	0
Encephalitis	1 (3)	0	1 (10)	0
Arthritis	1 (3)	0	0	1 (33)
Diabetes mellitus	1 (3)	0	1 (10)	0
IrAE ≥ during neoadjuvant phase	18 (58)	12 (67)	5 (50)	3 (100)
IrAE ≥ grade 3 during neoadjuvant phase †	7 (23)	4 (22)	1 (10)	2 (67)
AE related to surgery	8 (26)	5 (28)	0	3 (100)
AE related to surgery ≥ grade 3 †	3 (10)	3 (17)	0	0
Deaths related to treatment	1 (3)	0	0	1 (33)

* Percentages may not total 100 because of rounding; † Adverse events were graded using the NCI Common Terminology Criteria for Adverse Events (CTCAE), version 5.0. Abbreviations: adverse event, AE; immune-related AE, irAE.

## Data Availability

The original contributions presented in this study are included in the article. Further inquiries can be directed to the corresponding author.

## Abbreviations

The following abbreviations are used in this manuscript:

NADINA	Neoadjuvant trial, see references
SWOG S1801	Neoadjuvant trial, see references
EFS	Event-free survival
RFS	Recurrence-free survival
OS	Overall survival
DMFS	Distant metastasis-free survival
ICI	Immune checkpoint inhibitors
MPR	Major pathologic response
pCR	Pathologic complete response
pPR	Pathologic partial response
pNR	Pathologic non-response
FDG-PET	Fludeoxyglucose-18-positron emission tomography
SUV	Standardized uptake value
CMR	Complete metabolic response
PMR	Partial metabolic response
SMD	Stable metabolic disease
PMD	Progressive metabolic disease
IQR	Interquartile range
CI	Confidence interval
LDH	Lactate dehydrogenase
S-100	Tumor marker for melanoma
ULN	Upper limit normal
ITM	In-transit metastasis
CLND	Complete lymph node dissection
AE	Adverse event
ir	Immune-related
USZ	University Hospital Zurich, Switzerland
CTCAE	Common Terminology Criteria for Adverse Events

## Appendix A

### Appendix A.1

**Table A1:** Additional patients and treatment characteristics *.

Characteristics	Total Real-World Cohort *n* = 31	NADINA Real-World Cohort *n* = 18	SWOG 1801 Real-World Cohort *n* = 10	Mucosal Real-World Cohort *n* = 3
Mean time since primary melanoma—months (range)	16 (0–129)	20 (0–129)	12 (0–36)	0
ECOG performance status—no. (%) §				
0	19 (61)	15 (83)	3 (30)	1 (33)
1	8 (26)	3 (17)	4 (40)	1 (33)
2	4 (13)	0	3 (30)	1 (33)
S100 prior treatment—no (%)				
<ULN	19 (61)	11 (61)	7 (70)	1 (33)
>ULN	5 (16)	4 (22)	1 (10)	0
Not measured	7 (23)	3 (17)	2 (20)	2 (67)
LDH level prior treatment—no. (%)				
<ULN	19 (61)	14 (78)	4 (40)	1 (33)
>ULN	5 (16)	3 (17)	2 (20)	0
Not measured	7 (23)	1 (6	4 (40)	2 (67)
Mean time from staging to immunotherapy—weeks (range)	5 (3–11)	4 (3–10)	5 (3–11)	6 (4–7)
Number of neoadjuvant cycles—no. (%)				
1	2 (6)	1 (6)	0	1 (33)
2	19 (61)	17 (94)	0	2 (67)
3	10 (32)	0	10 (100)	0
Mean time from first cycle immunotherapy to surgery—weeks	8 (4–14)	6 (4–8)	10 (8–14)	10 (6–13)
Delayed surgery by toxicity—no. (%)	3 (10)	1 (6)	1 (10)	1 (33)
Performed surgery—no. (%)	28 (90)	16 (89)	9 (90)	3 (100)
Lymphadenectomy	15 (48)	10 (56)	4 (40)	1 (33)
Resection of in-transit or subcutaneous metastasis	3 (10)	1 (6)	2 (20)	0
Resection of the primary tumor	2 (6)	1 (6)	0	1 (33)
Lymphadenectomy and resection of the primary tumor	1 (3)	0	0	1 (33)
Lymphadenectomy and resection of in-transit or subcutaneous metastasis	4 (13)	1 (6)	3 (30)	0
Lymphadenectomy and re-excision with safety margins	3 (10)	3 (17)	0	0
Progression to unresectable disease prior to surgery	3 (10)	2 (11)	1 (10)	0
No adjuvant treatment after surgery and respective reason—no. (%)	12 (39)	6 (33)	4 (40)	2 (67)
Major pathologic response	8 (26)	5 (28)	3 (30)	0
Toxicity	3 (10)	1 (6)	1 (10)	1 (33)
Major pathologic response and toxicity	0	0	0	1 (33)
Completed all cycles of adjuvant treatment—no. (%)	2 (6)	1 (6)	1 (10)	0
Ongoing adjuvant treatment, no. (%)	10 (32)	6 (33)	4 (40)	0
Premature termination of adjuvant treatment and respective reasons—no. (%)	4 (13)	3 (17)	0	1 (33)
Recurrence locally	1 (3)	1 (6)	0	0
Progression to stage IV disease	1 (3)	1 (6)	0	0
Toxicity	2 (6)	1 (6)	0	1 (33)
Adjuvant treatment despite major pathologic response ‡	2 (6)	0	2 (20)	0

Abbreviations: Lactate dehydrogenase, LDH; Upper limit of normal, ULN. * Percentages may not total 100 because of rounding. § ECOG performance status scores range from 0 to 5, with higher scores indicating greater disability; a score of 0 indicates that the patient is fully active, 1 that the patient is restricted in strenuous activity but is ambulatory, and 2 that the patient is unable to work but is ambulatory and capable of self-care and up and about more than 50% of waking hours. ‡ Initially, all SWOG 1801 patients received adjuvant pembrolizumab irrespective of response, according to the SWOG 1801 protocol. Based on the results of the NADINA trial, the administration of adjuvant therapy was likewise adapted according to pathological response from the beginning of 2025.

### Appendix A.2

**Table A2:** Correlation of pathologic and radiologic response.

	CMR	PMR	SMD	PMD	NE	Summary
pCR	6	3		1	1	11
Near-pCR			1			1
pPR	1	1	1	3	1	7
pNR	1	1	4	2	1	9
Progression prior to surgery				2	1	3
Summary	8	5	6	8	4	31

Abbreviations: pCR, pathologic complete response; pPR, pathologic partial response; pNR, pathologic non-response. CMR, complete metabolic response; PMR, partial metabolic response; SMD, stable metabolic disease; PMD, progressive metabolic disease; NE, not evaluable (melanoma manifestation not measurable by radiologic imaging).
